# Resolving the Limitations of the CNI Model in Moral Decision Making Using the CAN Algorithm: A Methodological Contrast

**DOI:** 10.3390/bs12070233

**Published:** 2022-07-14

**Authors:** Chun Feng, Chuanjun Liu

**Affiliations:** 1 Department of Applied Psychology, Faculty of Law, Southwest University of Science and Technology, Mianyang 621010, China; fengchun@swust.edu.cn; 2 Department of Sociology and Psychology, and Institute of Psychology, School of Public Administration, Sichuan University, Chengdu 610065, China

**Keywords:** CAN algorithm, CNI model, methodological contrast, foreign language effect, moral decision making

## Abstract

The CNI model generates *C*, *N*, and *I* parameters to measure people’s mental processes—consequence sensitivity (*C*), norm sensitivity (*N*), and generalized inaction/action preferences (*I*)—in moral decision making. Given the limitations of the CNI model, the CAN algorithm was developed to depict the consequence sensitivity (*C*), overall action versus inaction preferences (*A*), norm sensitivity (*N*), and perverse responses with the other three parameters. However, no studies have clarified whether and how the CAN algorithm can solve the limitations of the CNI model. The present study systematically uncovers the limitations of the CNI model and the solutions provided by the CAN algorithm: (a) the CNI model does not consider negative values of the parameters, but the CAN algorithm does; (b) the sequential processing assumption of the CNI model is biased, the CAN algorithm proposes a parallel calculation strategy to fix this problem; (c) the calculation of the *I* parameter of the CNI model is inaccurate, so the CAN algorithm proposes the *A* parameter to replace it; (d) the CNI model has a problem measuring perverse responses, while the CAN algorithm develops three parameters to measure these. We examined some of our points on the basis of a reanalysis of the foreign language effect (FLE) by comparing the parameters from the CAN algorithm with those from the CNI model. We found that consequence and norm sensitivity were estimated to be greater using the CNI model than with the CAN algorithm. Consequently, these overestimations significantly (consequence sensitivity) and marginally (norm sensitivity) interfered with the FLE, making the FLE more likely to return a false positive result. In addition, the CAN algorithm was able to measure the extent of perverse responses, indicating that foreign language (compared to a native language) leads to more perverse responses. The present study demonstrates that the CNI model magnifies the Type I error of conclusions and that the CAN algorithm (compared to the CNI model) provides more insights regarding moral decision making.

## 1. Introduction

Would you kill one person to save five? Such decisions are difficult because the moral dilemmas entail a conflict between the deontological principle, which prohibits certain actions regardless of the consequences (e.g., hurting others violates the no-harm principle; thus, the action is unacceptable [1]) and the utilitarian principle, which is concerned with benefiting the greatest number of people (e.g., if more harm is prevented, hurting one person is acceptable [2].

There are three potential mental processes when participants make the moral decisions [3,4]. For example, they care about the utilitarian principle, showing consequence sensitivity, or they care about the deontological principle, showing norm sensitivity, or they can be generalized as having preferences for action over inaction, irrespective of norm and consequence. These three mental processes can be measured by three parameters, *C*/*N*/*I*, respectively. Thus, the model is referred to as the CNI model [4]. However, critics note that the CNI model is biased because of its theoretical [5,6] and methodological limitations [7]. To overcome the CNI model’s limitations, Liu and Liao [7] developed a new algorithm to depict consequence sensitivity (the *C* parameter), overall action versus inaction preferences across all kinds of scenarios (the *A* parameter), norm sensitivity (the *N* parameter), and another three parameters, referred to as the CAN algorithm. However, Liu and Liao [7] did not directly compare these two methods. It is unclear whether and how the CAN algorithm could solve the limitations of the CNI model. To fill in the gap, the present study has two aims.

First, the limitations of the CNI model and the solution of the CAN algorithm are discussed: (a) the CNI model does not consider negative values of the parameters, but the CAN algorithm does; (b) the sequential processing assumption of the CNI model is biased, while the CAN algorithm proposes a parallel calculation strategy to fix it; (c) the calculation of the *I* parameter of the CNI model is inaccurate, while the CAN algorithm proposes the *A* parameter to replace it; (d) the CNI model has a problem with perverse responses [5,6], while the CAN algorithm develops three parameters (*OI*, *OA*, and *MO*) for measuring perverse responses.

Second, we examined some of our points with a reanalysis of the FLE data [8]. The FLE refers to the notion that foreign language systematically influences the way people respond to moral dilemmas [1]. Białek and his colleagues [8] using the CNI model, found that foreign languages (compared to native languages) weaken sensitivity to both consequences and norms but do not affect generalized inaction/action preferences. Because of the limitations of the CNI model, the observed FLE would be not convincing in the CNI model. Thus, we directly compared the parameters from the CAN algorithm with those from the CNI model by reanalyzing previous data (e.g., FLE) to verify the applicability and reliability of the CNI model and the CAN algorithm. Before addressing our points in detail, we introduce the CNI model and the CAN algorithm, in turn.

### 1.1. The CNI Model and the CAN Algorithm

The CNI model is named after the abbreviations of the three parameters: (1) The *C* represents sensitivity to consequences (captured by the parameter *C*), which refers to how much people make their moral decisions based on the consequences of the proposed action, that is, whether the benefits of the action are greater or lesser than the costs. For example, people would tend to agree with the proposed action if it could result in greater benefits than costs, such as sacrificing one worker to save five. In contrast, they would not agree with the proposed action if it would lead to less benefits than costs, such as saving one person but sacrificing five. (2) The *N* represents sensitivity to norms (captured by the parameter *N*), which refers to the extent to which people make their moral decisions based on the moral norms underlying the proposed action, that is, whether the action is prohibited or advocated by moral norms. For example, people would tend to agree with the proposed action if the behavior is required by moral norms, which exist in most societies, such as gratitude, honesty, integrity, and kindness. In contrast, people would not tend to agree with the proposed action if the behavior was prohibited by moral norms, which exist in most societies, such as forbidding murder, rape, and torture. Finally, (3) the *I* represent a general preference for inaction over action, regardless of the consequences and norms (captured by the parameter *I* [4,9]. For example, if people do not care about consequences or norms, they will generally respond all ‘yes’ or all ‘no’ to the scenarios, ignoring the underlying principles related to consequences and norms.

**Figure 1:**
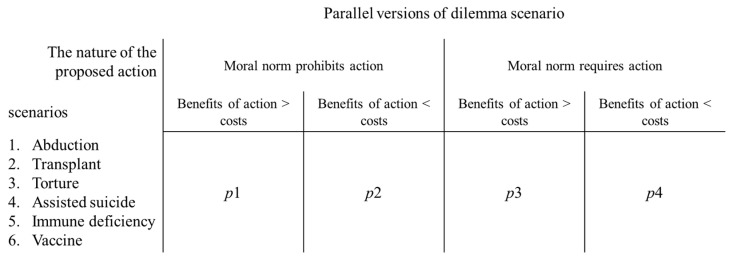
CNI model with dilemma factorials. Note. For each of the four parallel versions of dilemma scenarios, *p*1, *p*2, *p*3, and *p*4 indicate how likely people are to favor the corresponding action. In the CAN algorithm, the observed data from these four parallel versions of the dilemma scenario will be used.

On the basis of the combination of norms (required/prohibited) and consequences (the benefits of the action are greater/lesser than the costs; see Figure 1), the CNI model extends the classical moral dilemmas (kill one person to save five) to four types of scenarios: (1) dilemmas involving prohibited norms that prohibit action, where the benefits of the action are greater than the costs; (2) dilemmas involving prohibited norms that prohibit action, where the benefits of the action are less than the costs; (3) dilemmas involving required norms that require action, where the benefits of the action are greater than the costs; and (4) dilemmas involving required norms that require action, where the benefits of the action are less than the costs [3,4]. People choose whether to take action in these four types of scenarios. Recent studies using the CNI model have focused on moral decisions based on these four types of dilemma scenarios.

Although the CNI model has made a great contribution to our understanding of moral decisions, critics suggest that the CNI model is biased because of its theoretical [5,6] and methodological limitations [7]. To overcome these limitations, Liu and Liao [7] developed a new approach to address these concerns, named the CAN algorithm, which applies an algebraic method for generating the parameters, as shown in Table 1. This is fundamentally different from the maximum likelihood estimation of the CNI model.

The CAN algorithm is named for the abbreviation of its three parameters: The letter *C* represents sensitivity to consequences (captured by the *C* parameter) and the letter *N* represents sensitivity to norms (captured by the *N* parameter). The definitions of *C* and *N* in the CAN algorithm are identical to those in the CNI model. Specifically, the *C* parameter refers to the probability that the principle of consequence drives decisions. The greater the value of the *C* parameter, the more the people care about the consequence. The *N* parameter refers to the probability that moral norms drive decisions. The greater the value of the *N* parameter, the more the people care about moral norms.

The CAN algorithm introduced the *A* parameter to replace the *I* parameter of the CNI model. The letter *A* represents the extent of overall approval versus disapproval regarding the proposed actions. For example, in the trolley car dilemma, no matter how many workers are on the main and side tracks, and no matter whether taking action would kill workers, the participants do not care, and are generally approving of action. The *A* parameter measures this inclination. The greater the *A* parameter is, the greater the overall action bias. The other three parameters, *OI*, *OA*, and *MO*, were mentioned in Liu and Liao [7] and applied in their subsequent studies [7,10]. As shown in Table 1, the *OI* parameter depicts the extent to which people give inaction responses when both consequence and norm principles require action. For example, taking action will save five persons in the main track and no one will be harmed; however, people refuse to act. In contrast, when both consequence and norm principles forbid action, people might give perverse responses of action, which can be measured by the *OA* parameter. For example, taking action will save one person in the main track but kill five persons on the side track; nevertheless, people choose to act. In addition, the *MO* parameter describes the extent to which people are willing to follow the consequence and norm principles designed by the researcher, which could measure perverse responses as a whole. The lower the value of the *MO* parameter, the greater the number of total perverse responses. For example, people choose to take action or not based entirely on the requirements of the consequence and norm principles, as the researchers intended.

In summary, compared to the CNI model, the CAN algorithm proposes six parameters with the aim of being more nuanced and picturing the moral inclinations. It arithmetically requantifies parameters on the basis of the data of the CNI model. *p*1, *p*2, *p*3, and *p*4 are empirically observed probabilities (as shown in Figure 1).

### 1.2. Criticisms of the CNI Model and the Solutions of the CAN Algorithm

#### 1.2.1. The CNI Model Does Not Consider Negative Values of the Parameters, but the CAN Algorithm Does

The parameters estimated by the CNI model vary between 0 and 1, with no negative values [4]. However, it should be noted that people might choose options opposed to the requirements of moral principles.

Why might people choose the opposed options? There are many possibilities for this response pattern. First, people may not be able to follow the switching of perspective. In the scenarios of Gawronski and his colleagues [4], dilemmas are created by switching from one action, representing a harmful action on the part of the decision maker (e.g., a surgeon harvesting organs from a patient for other needy patients) to another in which the decision maker prevents another person from performing a harmful action (e.g., preventing another surgeon from killing a patient for the same reason). This switching may have an adverse effect on the decision maker [5], leading him or her to retaliate against the contravening party or to act against the rules because they may not want to take their responsibility seriously [11]. Second, people may not finish reading the decision carefully enough and may just answer the questions randomly. Random error could also lead to the opposed actions being selected.

This kind of opposite response should be represented by negative values. For example, people are more likely to approve of actions when the benefits of the action are less than the costs. In contrast, people are less likely to approve of actions when the benefits of the action are greater than the costs. In this case, the value of (*p*1 + *p*3) will be smaller than that of (*p*2 + *p*4). Furthermore, parameter *C* will be negative, as shown by the equation ($C=\frac{p1-p2+p3-p4}{2}$) in Table 1. However, the CNI model only codes the values of parameters as varying between 0 and 1, using the maximum likelihood estimation method. If these opposite responses are not coded as negative values, the parameter *C* will be overestimated in the CNI model. Similarly, the *N* parameter will also be overestimated, as no negative values are estimated.

The parameter generation strategy of the CAN algorithm differs from that of the CNI model. Specifically, the CNI model uses maximum likelihood estimation to generate the parameters, but it does not calculate negative values. In contrast, the parameters in the CAN algorithm are generated through algebraic operations, but not maximum likelihood estimation. The parameter generation strategy of the CAN algorithm is the subtracting and averaging method. This guarantees that negative values can be estimated. For example, when people are more likely to approve of the actions in a situation in which the benefits of the action are less than the costs, and when they are less likely to approve of actions in situations where the benefits of the action are greater than the costs, their consequence sensitivity will be calculated as a negative value in accordance with the equation for the *C* parameter presented in Table 1. Similarly, when people choose opposite responses from those required by the norm, the *N* parameter will also be calculated as a negative value. In this way, the *C* and *N* parameters will not be overestimated by the CAN algorithm.

#### 1.2.2. The Sequential Processing Assumption of the CNI Model Is More Likely to Overestimate the N Parameter, Whereas the Parallel Calculation Strategy Proposed by the CAN Algorithm Could Solve the Overestimation Issue

The CNI model presupposes that the agent must consider consequences, norms, and generalized inaction/action preferences in sequence. According to the corrective dual-process theory [12], the sequential processing logic of the CNI model leads to “*conditional relations of the parameters in determining behavioral outcomes*” [13]. Specifically, “*the processes underlying deontological judgments influence outcomes only if they are not overridden by the corrective processes underlying utilitarian judgments*” [13]. As shown in Table 1, the equation of the *N* parameter in the CNI model needs to be further divided (1−*C*). However, if the *N* parameter is estimated prior to the *C* parameter, the equation of the *N* parameter will not divide (1−*C*). The value of (1−*C*) is a decimal varying between 0 and 1. Consequently, the sequential processing in the CNI model will lead to the overestimation of the *N* (norm sensitivity) parameter [7].

In addition, Baron and Goodwin [6] also indicated the logic problem with the CNI model. If an experiment only manipulates either consequences, such as by changing the number of victims [14], or norms, such as by changing the directness of the killing [15], such manipulations are likely to affect the responses to the incongruent dilemmas. Specifically, if *C* comes first, the effects will almost exclusively be attributed to that, and vice versa if *N* comes first. Thus, the sequential processing used in the CNI model is problematic.

In contrast, the CAN algorithm proposes that the agent considers the consequences and norms simultaneously [7]. If people considered what they cared about sequentially, they would not feel that they were in a paradoxical situation. Taking the sequential processing pattern of the CNI model as an example, if the people sequentially consider consequences–norms–generalized inaction/action, they will only have one possibility (e.g., consequence) in mind at any given moment. Consequently, they will not sense the contradictions of the moral decisions. Thus, the ambiguity of thinking is evoked only when the consequences and norms in an incongruent dilemma are considered simultaneously.

To support this parallel processing logic and parameter calculation of the CAN algorithm, Liu and Liao [7] set up an alternative multinomial processing tree model. In a parallel processing manner, people’s moral decisions are driven by one of the norm or consequence principles, depending on which is stronger. This is more accurate than the CNI model for depicting the moral decision-making process, according to the literature [16,17].

**Figure 2:**
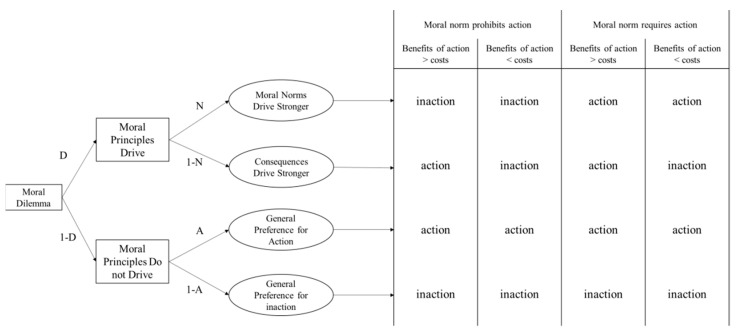
An alternative multinomial processing tree model constructed based on the corrective dual-process model of morality (the model can support the parallel processing logic and parameter calculation of the CAN algorithm). Note. *D* denotes that the agent’s choices are driven by moral principles (norms or consequences), and *N* denotes that the agent’s choices are driven more by norms. (1 − *N*) denotes that the agent’s choices are driven more by consequences. *A* denotes that the agent’s choices are driven by a preference for action, while moral principles do not drive their responses. According to the mechanism of the multinomial processing tree model, four equations are obtained, *p*1 = D × (1 − N) + (1 − D) × A; *p*2 = (1 − D) × A; *p*3 = D × N + D × (1 − N) + (1 − D) × A; *p*4 = D × N + (1 − D) × A. This figure was revised based on Liu and Liao [7].

The processing tree for depicting norm sensitivity would be the first branch on the left side of Figure 2 and can be calculated as $\frac{p3-p1+p4-p2}{2}$ on the basis of the equations. The processing tree for depicting the consequence sensitivity would be the second branch on the left side of Figure 2. It can be calculated as $\frac{p1-p2+p3-p4}{2}$ on the basis of the equation transformations. As can be seen, the equations are identical to the *C* and *N* parameters of the CAN algorithm presented in Table 1. Thus, the parallel calculation logic of the CAN algorithm is supported by the parallel processing tree model. As a result, the *N* parameter of the CAN algorithm will not be overestimated when compared to the *N* parameter of the CNI model.

#### 1.2.3. The Calculation of the I Parameter of the CNI Model Is Inaccurate; the CAN Algorithm Proposes the A Parameter to Replace It

Regarding the sequential processing logic in the CNI model, Liu and Liao [7] indicated that apart from the *C-N-I* processing sequence, there would be other processing sequences, such as *I-C-N* or *I-N-C*. This sequential processing logic will lead to the *I* parameter being unreliable due to the inaccuracy of its calculation. For example, when people first apply the general inaction or action response strategy and then consider whether they make choices according to their consequence and norm sensitivities, the denominator of *I* will not include [(1 − *C*) × (1 − *N*)] (see Table 1 for the equation). Thus, the calculation of the *I* parameter will be inaccurate.

Mathematically, as shown in Table 1, the calculation of the *I* parameter in the CNI model must assume that *C* and *N* should not be equal to 1. When people make moral choices totally in accordance with norm or consequence principles, *C* or *N* will be equal to 1. Consequently, the denominator of the *I* parameter will be 0, which in turn leads to the *I* parameter not being calculated accurately. Baron and Goodwin [6] also supported this idea. In contrast, the calculation of the *A* parameter in the CAN algorithm does not require this assumption (e.g., *C* or *N* could equal 1). Taken together, the *I* parameter is unreliable due to the inaccuracy of its calculation.

The CAN algorithm provides the *A* parameter, referring to the overall probability that people tend to accept behavioral proposals across the four parallel versions of the dilemma scenarios [7]. In contrast, the CNI model considered the overall extent of the refusal bias as the *I* parameter. Although theoretically the CNI model and the CAN algorithm focus on two different aspects of the same thing (overall extent of the action bias vs. overall extent of the refusal bias), the calculation in the CNI model is not reliable, as described above.

The *A* parameter in the CAN algorithm has an accurate logic of calculation. Specifically, if people follow the scenario set by the researchers exactly, they will completely refuse proposed actions that are forbidden by both consequence and norm principles. Thus, *p2* will be equal to 0. In contrast, they will completely approve of proposed actions that are required by both consequence and norm principles. Thus, *p3* will be equal to 1. With respect to the two incongruent scenarios, if the people fully understand the scenarios and the strengths of consequence and norm are equivalent, they will approve and disapprove of the proposed action, half and half, because of the ambiguity of the principles of consequence and norm. Thus, *p1* and *p4* will be equal to 0.5. Therefore, on the basis of the average values above, the *A* parameter will be equal to 0.5. However, if the people have not considered the consequences and norms, they will generally tend toward action or inaction in all versions of the scenarios, making the *A* parameter greater or smaller than 0.5. Taken together, the calculation equation and the meaning of the *A* parameter in the CAN algorithm are clearer than those of the *I* parameter in the CNI model, indicating that the *A* parameter in the CAN algorithm could replace the *I* parameter in the CNI model.

#### 1.2.4. The CNI Model Has a Problem with Perverse Responses, While the CAN Algorithm Develops Three Parameters (OI, OA and MO) for Measuring Perverse Responses

Baron and Goodwin [6] indicated that the design of the CNI model called for a large number of “perverse” responses to congruent situations, i.e., where both consequences and norms favor action, or where neither favor action. Such perverse responses are likely the result of interpreting the moral dilemmas differently from the way in which the experimenters intended. For instance, in case 2 of Gawronski et al’s work [4], the “transplant dilemma scenario”, where neither the consequences nor the norms favor action—do not kill a comatose patient who is going to die anyway for the purpose of transplanting organs into five other people who have unspecified “health problems”—people may respond in a perverse way (i.e., favoring action) for either of the following reasons: the norm against killing comatose patients who will die anyway is weak (particular when the action would save more people), or it is generally thought that health problems that must be treated with organ transplants must be quite serious. There is ambiguity about which norms apply and which consequences are worse, so people may respond perversely to congruent situations.

Specifically, Baron and Goodwin [6] proposed that the perverse responses occur because the scenario materials are ambiguous with respect to consequences and norms in the researchers’ designs. In consequence designs, the perverse responses occur in scenarios where both norm and consequence principles forbid the proposed action because people do not believe that the consequences designed by the researchers mean that the benefits of the action are less than its costs; thus, they approve of the action. In norm designs, perverse responses occur in scenarios where both the norm and consequence principles advocate for a proposed action because people do not believe that the norms designed by the researchers are advocatory rather than prohibitive; thus, they disapprove of the action. To conclude, when people face the ambiguity of scenario materials in consequence and norm designs, they might not agree with the assumptions of the researcher’s design.

These perverse responses might always exist because it is difficult to guarantee that people will precisely follow a researcher’s intentions. The CNI model does not consider how to solve this issue mathematically. The CAN algorithm suggests that a better way is to develop some new parameters for measuring how strong these ambiguities are in consequence and norm design and to try to control these perverse responses. To this end, the CAN algorithm developed three parameters (*OI*, *OA*, and *MO*) as indicators for measuring perverse responses. As shown in Table 1, the *OI* parameter describes the extent to which people do not accept the proposed action when both norm and consequence principles require them to accept the proposed action. For example, when people are expected only to help others because most people would benefit from their helpful behavior, they did not help others. In contrast, the *OA* parameter describes the extent to which people accept the proposed action, even though both norm and consequence principles require them to refuse the proposed action. For example, when people are expected to not harm others because most people would suffer from their harmful behavior, they nevertheless harm others. We assume that some people may select ‘inaction or action’, not only because they rely on consequences or norms but also because they may not understand the moral dilemma, particularly in the case of people who are reading the moral dilemma in a foreign language. The higher the value of the *OA* and *OI* parameters, the lower the inclination of people to follow either norm or consequence principles, and vice versa, indicating the observation of a stronger perverse response.

In addition, the CAN algorithm quantifies the extent to which people are following the norm and consequence principles in the scenarios designed by researchers. This is represented by the *MO* parameter. When the two principles require them to accept, they accept; when the two principles require them to refuse, they refuse. The lower the value of the *MO* parameter, the lower the inclination is to follow both the norm and consequence principles, and vice versa, indicating the observation of a stronger perverse response.

### 1.3. The Present Study

As indicated above, the CAN algorithm overcomes the theoretical and mathematical limitations of the CNI model and develops additional parameters (*OI*, *OA*, and *MO*) to quantify perverse responses. To further clarify whether and how the CAN algorithm is able to overcome the limitations of the CNI model, we conducted a reanalysis of raw data of the FLE [8] by comparing the CAN algorithm with the CNI model.

On the basis of the first two limitations of the CNI model described above, we predicted that the consequence and norm sensitivity might be estimated to be higher by the CNI model than when using the CAN algorithm. Consequently, this overestimation might interfere with the FLE, making the FLE more likely to display a false positive in the CNI model than in the CAN algorithm (Hypothesis 1). We also predicted that the three parameters (*OI*, *OA*, and *MO*) developed in the CAN algorithm would be able to measure the extent of perverse responses, indicating that foreign language (compared to a native language) leads to a greater number of perverse responses (Hypothesis 2).

Notably, we primarily aimed to compare the validations of the CAN algorithm and the CNI model, rather than to make broad inferences regarding the impact of the FLE on moral judgement per se. Thus, the present reanalysis mainly illustrates the applicability and reliability of the CAN algorithm by examining its validation using the study area of the FLE.

## 2. Methods

Because the present study is a methodological contrast, we did not gather new data; instead, we used the data of Białek and his colleagues [8], which we downloaded from osf.io/fzmsa/. We preregistered our research plan (osf.io/xymu2). In the registration, we described that the raw data of Białek and his colleagues [8] would be used as an example to draw a methodological contrast between the CAN algorithm and the CNI model.

In addition, there is a lack of control over the level of language proficiency and level of understanding of the moral scenarios when evaluating FLE in Białek and his colleagues [8]. Indeed, the level of language proficiency and level of understanding of the moral scenarios in a foreign language influence moral decisions [1,18]. Thus, we controlled the level of language proficiency and level of understanding of the moral scenarios to measure whether the moral judgment differed between the foreign language condition and the native language condition.

### 2.1. Participants of Białek et al.’s Work 

Białek and his colleagues [8] recruited 670 linguistics students (555 females, 104 males, 11 missing sex data) from a series of lectures. There was no compensation for participation. Polish bilingual students were fluent in English, German, Spanish, or French. Participants were randomly assigned to one of two language conditions: their native language or foreign language. Thirty-six participants who scored less than 5 out of 10 on understanding the scenarios were excluded from the analysis (15 in the native language condition, 21 in the foreign language condition). Finally, 634 participants (*M*_age_ = 21.75, *SD*_age_ = 3.04) were included in the analysis: 520 females and 104 males (9 participants did not report their sex), with 312 participants in the native language condition and 322 in the foreign language condition.

### 2.2. Procedure and Materials of Białek et al.’s Work 

Białek and his colleagues [8] presented the experimental materials to half of the participants in their native language (i.e., Polish); the other half received the materials in a foreign language (English, German, Spanish, or French, depending on their level of fluency). Based on Gawronski et al. (2017), each set of paper-and-pencil materials contained 24 moral dilemmas that were presented in a fixed random order. In response to the given dilemmas, participants were asked whether they would take the action by answering yes or no. (In the Polish–English sample, participants indicated their responses on a six-point scale. Białek and his colleagues converted the responses into yes/no binary scores for their analyses.) Then, the participants evaluated their language proficiency and how well they understood the scenarios in the moral dilemma task on a 10-point rating scale. Participants in the foreign language condition took slightly longer than those in the native language condition.

### 2.3. Reanalysis Procedure of Present Study

Firstly, we computed the parameters based on both the CNI model and the CAN algorithm. The individual *C*, *N*, and *I* parameters from the CNI model were calculated using the template provided by Korner and his colleagues [19] with the program multiTree v 0.46 [20]. The six parameters *C*, *N*, *A*, *OI*, *OA*, and *MO* from the CAN algorithm [7,21] were also generated.

Then, we conducted an analysis in two parts with the above parameters using SPSS 23.0. In the first part, we aimed to test hypothesis 1. We wanted to make a comparison of the CNI model and the CAN algorithm. Thus, we conducted two mixed ANOVAs with the *C* or *N* parameter generation approach (the CNI model/the CAN algorithm) as a within-subject variable and the language (native/foreign) as a between-subject variable to examine whether the different methodologies interacted with the foreign/native condition on consequence and norm sensitivities. In the second part, we aimed to test hypothesis 2. We conducted MANOVA with the parameters (*C*, *N*, *A*, *OI*, *OA*, and *MO*) generated from the CAN algorithm as dependent variables, language (native vs. foreign) as factors, and the level of language proficiency and understanding of the moral scenarios as covariates. We wanted to re-evaluate the FLEs with the six parameters and to test whether there were significant differences with respect to perverse responses between the foreign and native language conditions.

## 3. Results

### 3.1. Part 1 Interaction between Method (CNI Model/CAN Algorithm) and Language (Foreign/Native) on the C and N Parameters

As there were only 24 trials for the moral decisions, this might result in the occurrence of estimation errors when generating individual levels of *C*/*N* parameters using the CNI model [4]. When we computed the individual parameters for each participant with the individual CNI model protocol provided by Korner his colleagues [19], we excluded some participants from further analysis on the basis of two criteria: (1) at least one error was found when analyzing the dataset for 23 participants; (2) the probabilities predicted by the CNI model deviated at least marginally from the empirically observed probabilities for 103 participants, ∆*G*^2^(1) ≥ 2.72, *p* ≤ 0.099 (six participants simultaneously met the above two criteria). Finally, 120 participants were excluded from the following analyses. (We only excluded the 120 participants from the analysis comparing the CNI model and the CAN algorithm on C and N parameters because the issue of model fit only occurred in the CNI model and not in the CAN algorithm. Thus, the subsequent analyses still included the 120 participants.)

**Figure 3:**
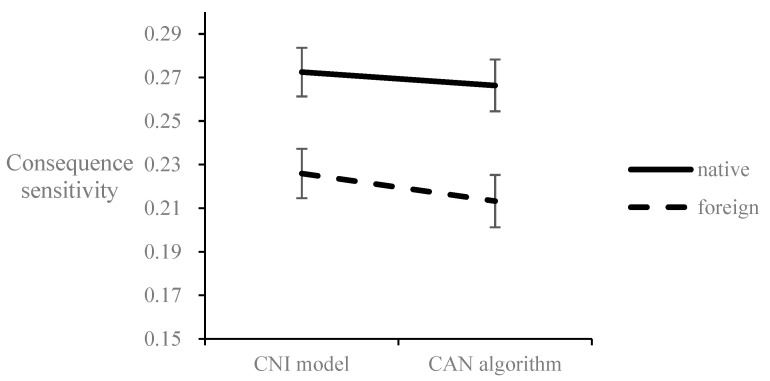
Consequence sensitivity estimated by the CNI model and the CAN algorithm significantly interacts with the foreign/native language condition. Error bar represents the ±1 standard error.

After filtering out the invalid data, we conducted two mixed ANOVAs with the *C* or *N* parameter generation approach (the CNI model/the CAN algorithm) as a within-subject variable and the language (native/foreign) as a between-subject variable. The results for the *C* parameter are shown in Figure 3. The *C* parameter for the CNI model (*M* = 0.25, *SE* = 0.01) is significantly greater than that for the CAN algorithm (*M* = 0.24, *SE* = 0.01), *F*(1,512) = 33.52, *p* < 0.001, η_p_^2^ = 0.06, indicating the overestimation of the *C* parameter in the CNI model compared to in the CAN algorithm. The *C* parameter in the foreign condition (*M* = 0.22, *SE* = 0.01) is significantly lower than that in the native condition (*M* = 0.27, *SE* = 0.01), *F*(1,512) = 9.30, *p* = 0.002, η_p_^2^ = 0.02, indicating an overall FLE on consequence sensitivity. The interaction effect is significant, *F*(1,512) = 4.08, *p* = 0.044, η_p_^2^ = 0.01. The simple effects showed that the overestimation of the *C* parameter generated using the CNI model compared to that generated using the CAN algorithm was even greater in the foreign condition (*M*_c-by-CNI_ = 0.23, *SE*_c-by-CNI_ = 0.01; *M*_c-by-CAN_ = 0.21, *SE*_c-by-CAN_ = 0.01; *F*(1, 512) = 30.15, *p* < 0.001, η_p_^2^ = 0.06) than in the native condition (*M*_c-by-CNI_ = 0.272, *SE*_c-by-CNI_ = 0.01; *M*_c-by-CAN_ = 0.266, *SE*_c-by-CAN_ = 0.01; *F*(1, 512) = 7.19, *p* = 0.008, η_p_^2^ = 0.01). The results indicated that consequence sensitivity estimated by the CNI model was overestimated compared to that estimated by the CAN algorithm. The overestimation of the consequence sensitivity was even greater in the foreign condition than in the native condition, thus significantly interacting with the FLE.

**Figure 4:**
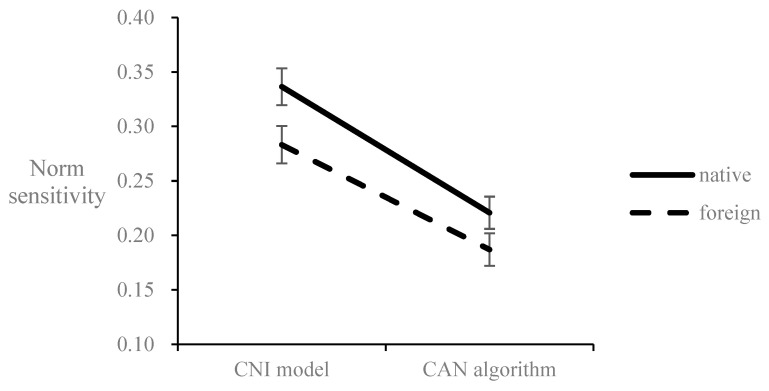
Norm sensitivity estimated by the CNI model and the CAN algorithm marginally interacts with the foreign/native language condition. Error bar represents the ±1 standard error.

The results for the *N* parameter are shown in Figure 4. The *N* parameter from the CNI model (*M* = 0.31, *SE* = 0.01) was significantly greater than that from the CAN algorithm (*M* = 0.20, *SE* = 0.01), *F*(1,512) = 431.56, *p* < 0.001, η_p_^2^ = 0.46, indicating the overestimation of the *N* parameter in the CNI model compared to the CAN algorithm. The *N* parameter in the foreign condition (*M* = 0.24, *SE* = 0.02) was significantly lower than that in the native condition (*M* = 0.28, *SE* = 0.02), *F*(1,512) = 3.92, *p* = 0.048, η_p_^2^ = 0.01, indicating an FLE on norm sensitivity. The interaction effect was marginally significant, *F*(1,512) = 3.61, *p* = 0.058, η_p_^2^ = 0.01. The simple effects show that the FLE is significant when using the *N* parameter from the CNI model (*F*(1,512) = 4.92, *p* = 0.027, η_p_^2^ = 0.01) and insignificant when using the *N* parameter from the CAN algorithm (*F*(1,512) = 2.58, *p* = 0.109, η_p_^2^ = 0.01). The results indicated that the norm sensitivity estimated by the CNI model was overestimated compared to that estimated by the CAN algorithm, and this overestimation magnified FLE at the marginal level.

### 3.2. Part 2 Re-Evaluate the FLE with the Parameters Determined Using the CAN Algorithm

Descriptive statistics are shown in Table 2. Given that the level of language proficiency and level of understanding of the moral scenarios (both were self-reported by the participants using a 10-point scale) in the foreign language condition influence moral decision [1,18], in the present study, we included these two variables in the subsequent analyses as covariates.

**Figure 5:**
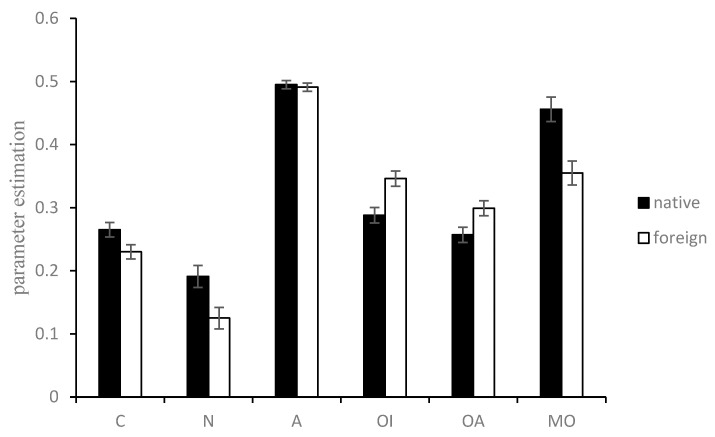
The parameter estimations using the CAN algorithm in the foreign/native language condition. Error bar represents the ±1 standard error. Note. Covariates appearing in the model are evaluated at the following values: proficiency = 6.74, understanding = 8.43.

To examine whether the different methodologies interacted with the foreign/native condition on consequence and norm sensitivities (As there were four pairs of native–foreign samples, we checked whether the different sample pairs had different FLEs. If not, the four samples could be combined to achieve greater statistical power [22]. We conducted MANOVA with the parameters *(C*, *N*, *A*, *OI*, *OA*, and *MO*) as dependent variables, samples (samples 1~4) and language (native vs. foreign) as factors, and the level of language proficiency and understanding of the moral scenarios as covariates. The results showed that the different samples did not interact with the FLEs on any of the six parameters (*F*(3,621) ≤ 1.90, *p* ≥ 0.129, η_p_^2^ ≤ 0.01). This means that there were no significantly different FLEs among the different samples. Thus, we combined the four samples to conduct the subsequent analysis), we conducted a MANOVA with the parameters (*C*, *N*, *A*, *OI*, *OA*, and *MO*) as dependent variables, language (native vs. foreign) as factors, and the level of language proficiency and understanding of the moral scenarios as covariates. The parameter estimations are shown in Figure 5, and the MANOVA results are shown in Table 3.

For the *C* parameter, participants in the foreign language condition (*M* = 0.22, *SD* = 0.20) had significantly lower consequence sensitivity than those in the native language condition (*M* = 0.27, *SD* = 0.20). For the *N* parameter, participants in the foreign language condition (*M* = 0.13, *SD* = 0.31) had significantly lower norm sensitivity than those in the native language condition (*M* = 0.18, *SD* = 0.28). For the *A* parameter, participants in the foreign language condition (*M* = 0.49, *SD* = 0.11) had statistically equivalent overall action bias to those in the native language condition (*M* = 0.50, *SD* = 0.11). For the *OI* parameter, participants in the foreign language condition (*M* = 0.34, *SD* = 0.21) had significantly greater generalized inaction preferences opposite to those required by norms and consequences than those in the native language condition (*M* = 0.29, *SD* = 0.20). For the *OA* parameter, participants in the foreign language condition (*M* = 0.30, *SD* = 0.21) had significantly greater generalized action preferences opposite to those required by norms and consequences than those in the native language condition (*M* = 0.26, *SD* = 0.20). For the *MO* parameter, participants in the foreign language condition (*M* = 0.36, *SD* = 0.35) had significantly lower moral obedience than those in the native language condition (*M* = 0.45, *SD* = 0.31) or more perverse responses.

In summary, on the basis of the two analyses, we found statistical evidence that the *C* and *N* parameters were estimated to be greater using the CNI model than with the CAN algorithm, and these overestimations significantly or marginally interacted with the foreign/native language condition.

When re-evaluating the FLEs using the parameters generated from the CAN algorithm, we found that when reading the dilemma in a foreign language (compared to a native language), participants might not have significant differences in terms of overall action bias, but they may exhibit a significant reduction in consequence sensitivity and norms sensitivity. However, these FLEs also have alternative explanations wherein foreign (compared to native) language leads to more perverse responses, specifically manifesting as a significantly higher inclination to make action and inaction choices opposite to those required by norms and consequences, and a significantly weaker inclination to follow the moral principles of norms and consequences.

## 4. Discussion

The present study provides a methodological contrast between the CAN algorithm and the CNI model by reanalyzing the raw data of FLE [8]. We found statistical evidence to support our methodological predictions. First, the *C* parameter from the CNI model is significantly overestimated, and the *N* parameter from the CNI model is overestimated at the marginal level compared to the CAN algorithm. Furthermore, the overestimations of the *C* and *N* parameters are even greater in the foreign condition than in the native condition. Thus, the CNI model magnifies the Type I errors on the *C* and *N* parameters, making the conclusions regarding these two parameters more likely to be false positives.

One possibility is that no negative values were generated by the CNI model. In contrast, a negative value was generated by the CAN algorithm. Consequently, the means of generating the *C* and *N* parameters would be better in the CNI model than in the CAN algorithm, particularly for participants in the foreign language condition, because they have less understanding of the moral scenarios compared to those in the native language condition. Indeed, participants might choose the options in contrast to the requirements of the moral principles when they did not understand moral scenarios very well, which should be coded by negative values. Thus, when the negative value was generated by the CAN algorithm, the *C* parameter from the CNI model was significantly overestimated, and the *N* parameter from the CNI model was overestimated by a marginal degree compared to the CAN algorithm. These findings supported our hypothesis 1.

In addition, the CNI model overestimates the *N* parameter because it assumes that the consequences principle prioritizes the norms principle when people make moral decisions. The CNI mode and the CAN algorithm have different *N* parameter equations (See Table 1). Specifically, the only difference between two equations is that the *N* parameter generated from the CNI model is further divided (1 − *C*) compared to the *N* parameter generated from the CAN algorithm. As the *C* parameter is a decimal value between 0 and 1, (1 − *C*) is also a decimal between 0 and 1. According to the rationale of division, any positive number divided by a decimal between zero and one will be larger than itself. Thus, the *N* parameter generated from the CNI model is estimated to be greater than that calculated using the CAN algorithm. More importantly, the CAN algorithm algebraically calculates the *N* parameter. The CAN algorithm is better aligned with the moral cognition literature because it allows for the independent calculation of both deontological and utilitarian preferences [15,16].

Second, we found that the participants who read moral dilemmas in a foreign language did not have significantly different scores on the overall action bias from those reading them in their native language. That is, the overall action bias probabilities did not differ between the foreign and native languages. Our findings exclude the possibility that the FLE can be attributed to an increase in the overall action bias in foreign languages compared to native ones.

Third, as two indicators for measuring perverse responses in the present reanalysis, we found that the participants had stronger inaction and action preferences that were opposed to norms and consequences when reading in a foreign language than in their native language. The *OI* and *OA* parameters represent the agent’s inaction and action preferences opposed to norms and consequences, respectively. These two parameters of the CAN algorithm presume that people sometimes do not follow the principles of norms and consequences. As shown in our results, the FLE could be found, potentially because the participants in the foreign language condition, compared to under the native language condition, were more likely to choose the options that were opposed to the requirements of both norm and consequence principles. One possibility is that more participants in the foreign language than in the native language condition might have a lower level of understanding of the moral scenario, leading to perverse responses.

Fourth, as another indicator for measuring total perverse responses in the present reanalysis, reading moral dilemmas in a foreign language (compared to a native language) leads to a reduction in the *MO* parameter. That is, participants who read the dilemma with a foreign language were less likely to follow both norm and consequence principles. These results might be because bilinguals who speak a foreign language are exempt from self- or socially imposed norms [23,24,25]. However, an alternative possibility is that participants in the foreign than native language condition might have a lower level of understanding of the moral scenario, leading to perverse responses. Thus, hypothesis 2 is supported. That is, the three parameters (*OI*, *OA* and *MO*) developed by the CAN algorithm were able to measure the extent of perverse responses, indicating that foreign language (compared to a native language) leads to more perverse responses.

Taken together, the CAN algorithm overcomes the limitations of the CNI model and provides a more nuanced perspective on the relationship between foreign languages and moral decision making. First, the most important contribution of the present study is that the CAN algorithm generates negative values and provides parallel processing of the estimated parameters, thus demonstrating deeper insight into the FLE. Specifically, the *C* and *N* parameters from the CNI model are overestimated compared to those estimated by the CAN algorithm, and this overestimation significantly or marginally magnified the FLE in the CNI model in comparison. The over-estimations of the *C* and *N* parameters make the FLEs on these two parameters more likely to be statistically significant. Thus, the type I error is magnified by the limitations of the CNI model.

Second, when participants interpret moral dilemmas differently from the experimenters’ intentions, perverse responses can occur [5]. The CNI model does not provide any solutions for perverse responses. However, the CAN algorithm developed three parameters (*OI*, *OA*, and *MO*) as indicators to measure the extent of perverse responses, thus providing a solution for perverse responses. Specifically, *OA*, *OI*, and *MO* can be used to measure three kinds of perverse responses: the *OI* parameter describes the extent to which participants do NOT accept the proposed action when both norm and consequence principles require them to accept the proposed action. In contrast, the *OA* parameter describes the extent to which participants accept the proposed action, even though both norm and consequence principles require participants to refuse the proposed action. In addition, the *MO* parameter describes the extent to which participants are following the norm and consequence principles in the scenarios designed by the researchers, thus indicating the overall extent of the perverse response observed.

This study has some limitations. First, as discussed by Baron and Goodwin [5,6], the manipulating strengths of consequence and norm may not be equivalent between different versions, and the participants might disagree with the scenario set by the researchers, which in turn results in perverse responses and biased parameter estimations. For example, the strength of the forbidden norm is generally stronger than the strength of the advocated norm; as a result, participants are more likely to disapprove of the proposed action in the forbidden versions than to approve of the proposed action in the advocated versions. In this situation, the *N* and *A* parameters would be underestimated, and the *C* parameter would be biased. The present study was not able to solve this issue because we did not gather new data, but rather reanalyzed previous raw data of the FLE. Thus, we could not revise the scenarios. This issue is worth further consideration in the future.

Second, although the CAN algorithm developed three parameters (*OI*, *OA*, and *MO*) as indicators to measure perverse responses, these parameters could not explain theoretically why the perverse response occurs. The present study found that participants reading moral dilemmas in a foreign language (compared to a native language) exhibited a reduction in the *MO* parameter and an increase in *OI* and *OA* parameters, revealing that a lower level of understanding of the moral scenario may lead to perverse responses. However, we did not collect original data and could not explore the role of understanding. The role of understanding is worth measuring in future studies.

Last, although the present study has demonstrated that the three parameters (*OI*, *OA*, and *MO*) could be indicators for measuring the perverse response, theoretically reducing the perverse responses would still be an issue. Baron and Goodwin [5,6] indicated that it would be possible to exclude these data related to the perverse response. Future studies should further discuss this issue by measuring the three parameters (*OI*, *OA*, and *MO*).

## 5. Conclusions

Consequence and norm sensitivity were estimated to be greater using the CNI model than with the CAN algorithm. Consequently, these overestimations significantly (consequence sensitivity) and marginally (norm sensitivity) interfered with the FLE, making the FLE more likely to return a false positive result. In addition, the CAN algorithm was able to measure the extent of perverse responses, indicating that foreign language (compared to a native language) leads to more perverse responses.

## Figures and Tables

**Table 1:** Equation contrasts between the CNI model and the CAN algorithm.

	CNI Model	CAN Algorithm
*C* parameter	$\frac{p1-p2+p3-p4}{2}$	$\frac{p1-p2+p3-p4}{2}$
*N* parameter	$\frac{p3-p1+p4-p2}{2\times \left(1-C\right)}$	$\frac{p3-p1+p4-p2}{2}$
*I* parameter	$\frac{1-p3}{\left(1-C\right)\times \left(1-N\right)}$	−
*OI* parameter	−	1−p3
*OA* parameter	−	p2
*MO* parameter	−	p3−p2
*A* parameter	−	$\frac{p1+p2+p3+p4}{4}$

Note. The equations of the CNI model are mathematically transformed from the equations provided by Gawronski et al.’s work [4]. The equations of the CAN algorithm are reported in Liu and Liao [7]. *p*1, *p*2, *p*3, and *p*4 are the probabilities of choosing to act in respective dilemmas as shown in Figure 1. *OI* means responses opposite to those required by consequences and norms. *OA* means action responses opposite to those required by consequences and norms. *MO* means moral obedience to both consequences and norms, which is the opposite of the total perverse responses.

**Table 2:** Mean estimates and standard deviations of parameters computed with the CAN algorithm.

Sample	Language	*n*	*C*	*N*	*A*	*OI*	*OA*	*MO*
Sample 1	Native: Polish	84	0.27 ± 0.19	0.19 ± 0.24	0.47 ± 0.13	0.33 ± 0.21	0.22 ± 0.20	0.45 ± 0.28
	Foreign: English	120	0.27 ± 0.18	0.11 ± 0.31	0.46 ± 0.11	0.36 ± 0.22	0.27 ± 0.20	0.37 ± 0.34
Sample 2	Native: Polish	75	0.21 ± 0.19	0.19 ± 0.25	0.50 ± 0.11	0.30 ± 0.20	0.30 ± 0.18	0.41 ± 0.29
	Foreign: German	63	0.19 ± 0.16	0.16 ± 0.33	0.51 ± 0.11	0.33 ± 0.21	0.32 ± 0.23	0.35 ± 0.36
Sample 3	Native: Polish	83	0.29 ± 0.19	0.18 ± 0.30	0.50 ± 0.09	0.28 ± 0.20	0.25 ± 0.20	0.47 ± 0.34
	Foreign: Spanish	80	0.22 ± 0.21	0.14 ± 0.31	0.51 ± 0.10	0.33 ± 0.22	0.31 ± 0.20	0.36 ± 0.36
Sample 4	Native: Polish	70	0.31 ± 0.23	0.17 ± 0.33	0.52 ± 0.10	0.25 ± 0.19	0.27 ± 0.20	0.48 ± 0.32
	Foreign: French	59	0.17 ± 0.22	0.15 ± 0.28	0.50 ± 0.11	0.36 ± 0.20	0.32 ± 0.23	0.32 ± 0.36
Combined	Native	312	0.27 ± 0.20	0.18 ± 0.28	0.50 ± 0.11	0.29 ± 0.20	0.26 ± 0.20	0.45 ± 0.31
	Foreign	322	0.22 ± 0.20	0.13 ± 0.30	0.49 ± 0.11	0.35 ± 0.21	0.30 ± 0.21	0.36 ± 0.35

**Table 3:** The MANOVA results in the combined sample (n = 634).

		*C*	*N*	*A*	*OI*	*OA*	*MO*
Native–Foreign	*F*	4.41 *	6.68 *	0.22	10.69 ***	5.60 *	12.59 ***
	*p*	0.036	0.010	0.641	0.001	0.018	0.000
	η_p_^2^	0.01	0.01	0.00	0.02	0.01	0.02
Language proficiency	*F*	3.31	2.86	1.08	0.22	0.04	0.18
	*p*	0.069	0.092	0.298	0.641	0.840	0.673
	η_p_^2^	0.01	0.00	0.00	0.00	0.00	0.00
Understanding of the moral scenarios	*F*	31.16 ***	0.07	0.13	4.03 *	8.51 **	9.56 **
	*p*	0.000	0.797	0.719	0.045	0.004	0.002
	η_p_^2^	0.05	0.00	0.00	0.01	0.01	0.02

Note: the language proficiency and the understanding of the moral scenarios are continuous variables, and the participants rated them with 10-point scales; * *p* < 0.05, ** *p* < 0.01, *** *p* < 0.001.

## Data Availability

No new data are reported in present study.
